# Measuring health-related quality of life in men with osteoporosis or osteoporotic fracture

**DOI:** 10.1186/1471-2458-11-775

**Published:** 2011-10-09

**Authors:** Marta Zwart, Rafael Azagra, Gloria Encabo, Amada Aguye, Genís Roca, Sílvia Güell, Núria Puchol, Emili Gene, Francesc López-Expósito, Silvia Solà, Sergio Ortiz, Pilar Sancho, Liz Abado, Milagros Iglesias, Jesus Pujol-Salud, Adolf Diez-Perez

**Affiliations:** 1 EAP Can Gibert del Plà- Girona-2 Health Centre, Catalan Health Institute. Carrer Sant Sebastià 50, 17005 Girona, Spain; 2 Department of Medicine, Universitat Autònoma de Barcelona. Passeig Vall d'Hebrón 119-129, 08035 Barcelona, Spain; 3 EAP Badia del Vallès Health Centre, Catalan Health Institute. Carrer Bética, 08214 Badia del Vallès, Barcelona, Spain; 4 Primary Healthcare Research Support Unit Metropolitana Nord. Catalan Health Institute-IDIAP Jordi Gol. Rambla 227, 08223 Sabadell, Barcelona, Spain; 5 Nuclear Medicine Service, Vall d'Hebron University Hospital. Passeig Vall d'Hebrón 119-129, 08035 Barcelona, Spain; 6 EAP Granollers-Centre Health Centre, Catalan Health Institute. Carrer Museu 19, 08400 Granollers, Barcelona, Spain; 7 EAP Sant Llatzer Health Centre. Consorci Sanitari de Terrassa. Carrer de la Riba, 62, 08221 Terrassa, Barcelona, Spain; 8 EAP Montcada i Reixach Health Centre, Catalan Health Institute. Passeig de Jaume I, 08110 Montcada i Reixac, Barcelona, Spain; 9 Emergency Service, Hospital of Sabadell, Sanitary Consortium of Parc Taulí. Parc Tauli, 08208 Sabadell, Barcelona, Spain; 10 EAP Bon Pastor Health Centre, Catalan Health Institute. Carrer de Mollerussa, 08030 Barcelona, Spain; 11 Emergency Service, University Hospital of Bellvitge, Catalan Health Institute. University of Barcelona. Carrer de la Feixa Llarga, 08907 L'Hospitalet de Llobregat, Barcelona, Spain; 12 EAP Serraparera Health Centre, Avda Diagonal, 08290 Cerdanyola del Vallès, Barcelona, Spain; 13 EAP Corbera de Llobregat Health Centre, Catalan Health Institute. c/Buenos Aires, 9, 08757 Corbera de Llobregat, Barcelona, Spain; 14 Internal Medicine Service, IMIM-Hospital del Mar. Department of Medicine. Psg Marítim 25, 08003 Barcelona. Spain; 15 EAP Balaguer Health Centre, Catalan Health Institute. Passeig Àngel Guimerà, 22, 25600 Balaguer. Lleida. Spain

## Abstract

**Background:**

Osteoporosis is a serious health problem that worsens the quality of life and the survival rate of individuals with this disease on account the osteoporotic fractures. Studies have long focused on women, and its presence in men has been underestimated. While many studies conducted in different countries mainly assess health-related quality of life and identify fracture risks factors in women, few data are available on a Spanish male population.

**Methods/Design:**

Observational study.

**Study population:**

Men ≥ 40 years of age with/without diagnosed osteoporosis and with/without osteoporotic fracture included by their family doctor.

**Measurements:**

The relationship between customary clinical risk factors for osteoporotic fracture and health-related quality of life in a Spanish male population. A telephone questionnaire on health-related quality of life is made.

**Statistical analysis:**

The association between qualitative variables will be assessed by the Chi-square test. The distribution of quantitative variables by Student's t-test. If the conditions for using this test are not met, the non-parametric Mann-Whitney's U test will be used.

The validation of the results obtained by the FRAX™ tool will be performed by way of the Hosmer-Lemeshow test and by calculating the area under the Receiver Operating Characteristic (ROC) curve (AUC). All tests will be performed with a confidence intervals set at 95%.

**Discussion:**

The applicability and usefulness of Health-related quality of life (HRQOL) studies are well documented in many countries. These studies allow implementing cost-effective measures in cases of a given disease and reducing the costly consequences derived therefrom. This study attempts to provide objective data on how quality of life is affected by the clinical aspects involved in osteoporosis in a Spanish male population and can be useful as well in cost utility analyses conducted by health authorities.

The sample selected is not based on a high fracture risk group. Rather, it is composed of men in the general population, and accordingly comparisons should not lead to erroneous interpretations.

A possible bias correction will be ensured by checking reported fractures against healthcare reports and X-rays, or by consulting health care centers as applicable.

## Background

### Epidemiology

The study of osteoporosis (OP) has long focused on women, and its presence in men has been underestimated despite the existence of important morbidity and mortality data.

Prevalence data in Spain reflect densitometric OP percentages of 6% in men aged between 60 and 69 years, 11.3% in the lumbar spine of men aged between 70-79 years, and 2.6% in the femoral neck of older men [1,2].

Epidemiological data of vertebral fractures in a Spanish male population are difficult to calculate because 30% of these are symptomatic; however, a prevalence rate of 572 per 100,000 population was published by the EVOS study [3]. Hip fractures are the easiest to document because most of them require hospitalization for treatment. Data from the Ministry of Health and Consumption [4] reports an annual incidence rate of these fractures among men of 270 cases per 100,000 people older than 64 years. Also to be emphasized is that the mortality rate after vertebral fracture seems to be higher in men as compared to women [5] in the older age groups.

### Health-related quality of life (HRQOL)

Decreased HRQOL in women with osteoporotic fracture is well documented, as are the economic repercussions involved (the occurrence of fractures implies a loss of 0.3 HRQOL points on the EQ-5D health state classification [1]). Also, this worsening persists for years after sustaining the fracture [2]. In contrast, little information is available in the male population. The purpose of this study is to objectively measure the degree of HRQOL worsening in men by validated questionnaires, complete with clinical aspects related to osteoporosis and bone density scan results (DXA).

OP-related HRQOL distinguishes between OP patients with or without fracture. These two categories worsen the patient's quality of life on account of fear to sustain new fall-related fractures, fracture-related pain, resulting vertebral deformity, respiratory compromise, limited hip mobility due to some fractures, and death of a relevant number of patients within one year after hip fracture surgery [6].

Patients may be evaluated by generic HRQOL instruments such as the SF-36 [7], the EuroQol [8], or the COOP/WONCA charts [9]. The latter, however, may lack sensitivity to detect significant clinical changes within the scope of OP, such as morbidity caused by vertebral fractures and resulting pain or inability, impaired body image, possible isolation, or mood alterations. Accordingly, specific questionnaires to evaluate OP have been created, such as the QUALEFFO (Quality of Life Questionnaire of the European Foundation for Osteoporosis) [10] or the ECOS-16 [11] questionnaires. A possible drawback of these specific questionnaires is the impossibility of making comparisons between different populations.

The EuroQol-5D (EQ-5D) is a generic HRQOL instrument that consists of 2 parts: one describes the different health dimensions and the other is a Visual Analogue Scale. The items assessed include mobility, self-care, daily activities, pain, and anxiety/depression. Each dimension is divided into 3 degrees of severity: none, moderate and severe (scored 1, 2 and 3, respectively). The order of dimensions must be the same at all times.

ECOS-16 is an OP-specific questionnaire validated in Spain, obtained from the generic SF-36 and the QUALEFFO questionnaires. It is a shorter questionnaire (16 items) and thus proves useful in the daily medical practice. The health state is divided into 4 dimensions: physical function (5 items), pain (5 items), fear of illness (2 items), and psychosocial function (4 items). Each dimension is divided into 5 degrees of severity, varying from 1 (best health state) to 5 (worst health state).

Both EQ-5D and ECOS-16 are readily administered questionnaires (< 5.0 minutes and 12.3 minutes, respectively) and accordingly these were selected for HRQOL evaluation in our study. Indeed, time is a major drawback when assessing the biopsychosocial context of our patients and, on the other hand, their briefness helps keep the patient's and the health care provider's interest awake.

### OP and osteoporotic risk factors for fracture in men

While many studies conducted in different countries identify fracture-related risk factors [12], few data are available on the Spanish male population.

In the year 2004, Kanis et al [13] identified low BMI, smoking, family history of fragility fracture and glucocorticoid intake as risk factors for fracture in men. Other risk factors cited in the literature [14,15] include old age (> 70 years), personal history of fracture, alcohol consumption, weight loss, lack of exercise and situations of decreased bone strength, such as androgen suppression, rheumatoid arthritis, diabetes mellitus, hypothyroidism, chronic obstructive pulmonary disease, symptoms of gastrointestinal malabsorption, and cardiovascular disease [16]; also, exposure to certain drugs other than typical corticosteroids, such as anti-androgen therapy, thyroid therapy, hypolipidemic drugs (statins), antidiabetic drugs (glytazones), and antacids (proton pump inhibitors [17]). The evidence for some of these is controversial.

There is consensus on the use of clinical risk factors for fracture in combination with bone mineral density (BMD) measurements in the evaluation and the decision-making process regarding OP patients. Reliable tools to evaluate the risk factor for fractures in both men and women, such as the FRAX™ algorithm designed by the WHO, are now available [18]. This tool has proved to be very valuable to detect densitometric osteoporosis in a Spanish population [19,20], and allows obtaining absolute 10-year probability of major or hip fracture by using clinical risk factors for fracture associated or not associated to bone density measurement by DXA. Recently, the evaluation of fracture risk factors other than those included in the FRAX™ is being advised [21]. This study attempts to update knowledge on this subject, with focus placed on a Spanish male population.

### Objectives

#### Primary objective

To determine the loss of health-related quality of life in men with osteoporosis or osteoporotic fracture in a Spanish population.

#### Secondary objective

To determine the relationship between questionnaire clinical risk factors for osteoporotic fracture (age, body mass index), personal and family history of fracture, comorbidity, risk of falls, pharmacological treatments, low bone mineral density) and health-related quality of life in a male population.

## Methods/Design

### Design

This was an observational study of a random sample of men included in a quota of primary care physicians from the 4 provinces of Catalonia to evaluate HRQOL in the scope of osteoporosis.

### Study population

Urban environment, primary care.

Men ≥ 40 years of age with/without diagnosed OP and with/without osteoporotic fracture who are asked by their family doctor (either during consultation or by telephone and by the e-CAP software [22] used in primary care centers attached to the Catalan Institute of Health) to answer a questionnaire on risk factors for osteoporotic fracture and on existing fractures, as well as a further telephone questionnaire on HRQOL.

### Sample size

Assuming a 0.35 deviation of EQ-5D, a difference in the means of the variables between fractured and non-fractured patients of 0.15 is detected in a sample of 280 individuals (140 fractured and 140 non-fractured individuals, statistical significance set at 5% and power of 95%).

### Sample screening

Random sample of men from the populations assigned to the participating primary care physicians from the 4 provinces of Catalonia, age- and sex-matched to the population of Catalonia.

### Inclusion criteria

Male individuals born and living in Catalonia, of Caucasian race, and aged ≥ 40 years and ≤ 90 years at the time of inclusion in the study (FRAX™ is calculated in populations aged 40-90 years). Men able to understand and express themselves in the Spanish or Catalan language, able to answer the questionnaire as written and agreeing to participate in the study by signing an informed consent form. Physically or mentally disabled subjects whose representative agrees to answer the questionnaires and gives consent on behalf of the subject.

### Exclusion criteria

Subjects aged < 40 years and/or > 90 years at the time of sampling. Subjects with physical or mental difficulties or limitations that prevent them from participating in the study as scheduled or with relatives who refuse to answer the questionnaires. Subjects with Paget's disease or bone cancer. Subjects refusing to complete the questionnaires during the clinical interview or failing to give their consent to answer the telephone questionnaire for any reason. Subjects failing to provide a contact telephone number, or failing to respond to 3 phone calls made at different times.

### Overview of outcome measurements

#### Data collection

The project will be initiated at primary care centers by physicians specializing in Family and Community Medicine. Field work will include completing a RFQ and ascertaining the existence of fracture at the clinic or by telephone (by the family doctor responsible for the participating subject) once verbal consent has been obtained and recorded. The participation of the different primary care investigating physicians will be coordinated and supervised by the primary care centers CAP Girona-2 and CAP Badía del Vallés, which will also gather the information collected in the different provinces, with data treatment performed by a statistician and a database manager. Upon the collection of the above information, the principal investigator of this study -also a physician specializing in Family and Community Medicine- will make phone calls from the CAP Girona-2 center to administer the HRQOL questionnaires.

This study has been authorized by the CREC of the Hospital Vall d'Hebron of Barcelona. Also, consent to participate in the study and to answer the questionnaires, as well as future contacts of the research team, will be gathered from electronic medical records.

Baseline variables and RFQs will be collected at the time of inclusion (2011). Current variables will be collected once all baseline variables from the participating individuals have been recorded (june 2011-december 2012).

### Baseline variables

• Patient-related variables:

○ Demographic variables: date of birth (dd/mm/yyyy).

○ Anthropometric variables: bodyweight (kg), height (cm), body mass index (BMI) (kg/m^2^).

○ Level of education: none/primary/secondary/higher education.

○ Employment status: employed/unemployed/retired/disabled.

• Clinical risk factors for fracture:

○ Family history of hip fracture (father/mother). Y/N

○ Medical history of diseases involving loss of bone strength. Y/N

○ History of fragility fracture (defined as fall from standing height or less). Y/N. Location and date will be recorded. Axial skeleton fractures (ribs, dorsal and lumbar vertebrae) and peripheral fractures (wrist, femur and humerus) will be evaluated.

○ Smoking. Y/N

○ Alcohol risk intake (> 3 AU/week). Y/N

○ History of glucocorticoids intake for at least 3 months and doses ≥ 5 mg/day in the past 2 years: Y/N.

○ Medical history of osteoporotic medication. Y/N. Name and number of months.

○ Number of falls over the past year. While different ways to analyze falls have been found in the literature, the most frequently used one was selected in our study, i.e. asking about the number of falls over the year before administering the questionnaire, and whether any of these falls resulted in fractures.

▪ Use of walking cane: Y/N

▪ Needs help to stand up from a chair: Y/N

• Previous Bone Density Scan by Dual-energy X-ray absorptiometry (DXA): Y/N. BMD measurement recording if present in the medical record. These values will be analyzed according to internationally accepted WHO criteria of 1994 and ISCD recommendations. (Available at: http://www.iscd.org/Visitors/positions/OfficialPositionsText.cfm).

• Percent values obtained by the FRAX™ calculation tool regarding absolute risk of major osteoporotic fracture (hip, humerus, forearm and clinical spine) and hip fracture. The variables are gathered in the RFQ and calculations are performed at the official website (http://www.shef.ac.uk/FRAX). All results are expressed in percentage (%) of absolute risk of sustaining a new (major or hip) fracture during the following 10 years. Individual calculations on a case-by-case basis are performed by two separate investigators. The results of each calculation are analyzed by another two investigators. The results are compared by another two investigators, and any difference found leads to risk recalculation (triple evaluation of risk calculated by FRAX).

### Follow up variables

HRQOL questionnaires.

• General (See annex 1: EQ-5D quality of life questionnaire).

• OP-specific (See annex 2: ECO-16 quality of life questionnaire).

### Type of analysis

The characteristics of the studied population will be described by univariate descriptive analysis with calculation of median, mean, standard deviation, maximum and minimum values, and percentages for categorical data. Bar charts and histograms will be used for graphical representation.

The association between qualitative variables will be assessed by the Chi-square test.

The differences in the distribution of a quantitative variable according to the categories defined for a binary qualitative character will be assessed by Student's t-test. If the conditions for using this test are not met, the non-parametric Mann-Whitney's U test will be used [23].

The differences in the distribution of a quantitative variable according to the categories defined by a qualitative variable with more than 2 categories will be assessed by analysis of variance (ANOVA) or the equivalent non-parametric test (Kruskal-Wallis) [23].

Bivariate comparisons between categorical variables (Chi-square test) and between quantitative and categorical variables (Student's t-test) will be performed to ascertain the age- and sex-related distribution of risk factors associated with sustaining a fracture.

The validation of the results obtained by means of the FRAX™ tool will be performed by way of the Hosmer-Lemeshow test and by calculating the area under the Receiver Operating Characteristic (ROC) curve (AUC).

The Hosmer-Lemeshow test [23] consists of dividing our individuals in k groups (usually 10) depending on their lower or higher fracture risk according to FRAX™, and checking that each group actually presents with a number of fractures that matches the FRAX™ prediction. Few cases are expected in the first (lower risk) group, with successive increases in the following groups.

All tests will be performed with a confidence interval set at 95%. Analyses will be performed with SPSS statistics software 16.0 or higher.

### Personal data protection

The investigators guarantee and hold themselves responsible for data confidentiality. All data are entered into a computer database where patients are identified by a code. A parallel database is created which includes the particulars of each participant and the related code. This database can only be accessed by the Principal Investigator, the Coordinating Investigator and the Computer Technician.

## Discussion

The applicability and usefulness of HRQOL studies are well documented in many countries. These studies allow implementing cost-effective measures in cases of a given disease and reducing the costly consequences derived therefrom. This study attempts to provide objective data on how quality of life is affected by the clinical aspects involved in osteoporosis in a Spanish male population. The studies so far conducted in men included small populations. The purpose of this study is to evaluate a larger population -representative of the general population- and thus obtain generalizable results and data likely to be useful in cost utility analyses conducted by health authorities.

The publication in 2008 of the FRAX tool to calculate 10-year absolute probability of fragility fractures offers a new approach to patients at higher risk of sustaining a fracture. In the pilot test where the FRAX™ fracture risk calculator was used in 100 real cases, errors between 1-3% were detected in each case. The analysis plan with the FRAX™ calculator will include double analysis performed by several investigators blinded to calculation results. Any and all differences will be evaluated by a third investigator.

Our study has several strengths and limitations. The HRQOL questionnaires selected are to be self-administered, that is, completed by the patients themselves. Because these questionnaires will be administered by telephone by a well-documented, well-trained physician, we hope to reduce the limitations specific to this data collection system [24].

The ECOS-16 questionnaire has been validated in women. In our opinion, however, this does not invalidate the results of the study because no sex-specific questions or answers were envisaged.

The sample selected is not based on a high fracture risk group. Rather, it is composed of men in the general population, and accordingly comparisons should not lead to erroneous interpretations.

Data collection may involve biased information on incident fractures, it being obtained according to the patient's account (no X-ray tests). Consequently, a small percentage of (basically vertebral) silent fractures may go undetected. Nevertheless, this is a questioner, internationally accepted means of ascertaining the incidence of fractures in epidemiological studies, known as self-reported related fractures. Bias correction will be ensured by checking reported fractures against healthcare reports and X-rays, or by consulting health care centers as applicable.

## List of abbreviations

RFQ: Risk Factor Questionnaire; HRQOL: Health-Related Quality Of Life; DXA: Dual-Energy X-Ray Absorptiometry; BMD: Bone Mineral Density; EQ-5D: EuroQol-5D; EVOS: European Vertebral Osteoporosis Fracture Study; FRAX™: Fracture Risk Assessment tool; BMI: Body Mass Index; ISCD: International Society for Clinical Densitometry; OP: Osteoporosis; Qualeffo: Quality of Life Questionnaire of the European Foundation for Osteoporosis; Y: Yes; N: No; AU: Alcohol units.

## Competing interests

This manuscript has not been wholly or partially submitted elsewhere and has been read and approved by all the authors and there is no conflict of interest.

In 2009 the protocol was granted a 3,000 Euros fellowship to the Best Doctoral Thesis Project of the Universitat Autònoma de Barcelona-Novartis Chair in Family Medicine Teaching and Research.

In 2010 the protocol was granted a fellowship on Research Training and Doctorate (PhD development) in Primary Care of the IDIAP Jordi Gol i Gurina. Throughout a two years period of the grant, 25% of working time will be devoted to the completion of the PhD.

The study has been approved by the Ethics Committee of the Research Institute of Vall d'Hebron University Hospital, Barcelona, Spain. *(IR Hospital de la Vall d'Hebron. Universitat Autònoma de Barcelona, Barcelona)*

The investigators guarantee and hold themselves responsible for data confidentiality. All data are introduced into a computer database where patients are identified by a code. A parallel database is created which includes the particulars of each participant and the related code. This database can only be accessed by the Principal Investigator, the Coordinating Investigator and the Computer Technician.

## Authors' contributions

MZ and RA: project design and management, manuscript review. GE: DXA-scan process coordination, manuscript review. AA: field work coordination and manuscript review. GR, SG, NP, FLE, SS, SO, MPS, LA, MI: field work, calculation of FRAX™ values and manuscript review. PT: manuscript review coordination. EG and ADP: scientific support and methodological expertise, manuscript review.

All authors have read and approved the final manuscript.

## Appendices

### Annex 1: EQ-5D QUALITY OF LIFE QUESTIONNAIRE

Under each heading, please tick the ONE box that best describes your health TODAY.

#### Mobility

I have no problems in walking about ❏

I have slight problems in walking about ❏

I have moderate problems in walking about ❏

I have severe problems in walking about ❏

I am unable to walk about ❏

#### Self-Care

I have no problems washing or dressing myself ❏

I have slight problems washing or dressing myself ❏

I have moderate problems washing or dressing myself ❏

I have severe problems washing or dressing myself ❏

I am unable to wash or dress myself ❏

#### Usual activities *(e.g. work, study, housework, family or leisure activities)*

I have no problems doing my usual activities ❏

I have slight problems doing my usual activities ❏

I have moderate problems doing my usual activities ❏

I have severe problems doing my usual activities ❏

I am unable to do my usual activities ❏

#### Pain/Discomfort

I have no pain or discomfort ❏

I have slight pain or discomfort ❏

I have moderate pain or discomfort ❏

I have severe pain or discomfort ❏

I have extreme pain or discomfort ❏

#### Anxiety/depression

I am not anxious or depressed ❏

I am slightly anxious or depressed ❏

I am moderately anxious or depressed ❏

I am severely anxious or depressed ❏

I am extremely anxious or depressed ❏

### Annex 2: ECOS-16 QUALITY OF LIFE QUESTIONNAIRE

1. How often have you had back pain in the last week?

☐ 1. I have had no back pain *(proceed to question no 6)*

☐ 2. 1 day

☐ 3. 2-3 days

☐ 4. 4-6 days

☐ 5. Every day

2. How severe was your back pain?

☐ 1. Mild

☐ 2. Annoying

☐ 3. Moderate

☐ 4. Severe

☐ 5. Intolerable

3. How much distress or discomfort have you had due to pain from standing for a long time?

☐ 1. No discomfort or suffering

☐ 2. Slight discomfort or suffering

☐ 3. Moderate discomfort or suffering

☐ 4. Severe discomfort or suffering

☐ 5. Very severe discomfort or suffering

4. How much distress or discomfort have you had due to pain from bending?

☐ 1. No discomfort or suffering

☐ 2. Slight discomfort or suffering

☐ 3. Moderate discomfort or suffering

☐ 4. Severe discomfort or suffering

☐ 5. Very severe discomfort or suffering

5. Has back pain disturbed your sleep in the last seven days?

☐ 1. On no occasion

☐ 2. One night

☐ 3. Two nights

☐ 4. Three or four nights

☐ 5. Every night

6. How difficult has it been for you to carry out household activities?

☐ 1. No difficulty

☐ 2. Slight difficulty

☐ 3. Moderate difficulty

☐ 4. Great difficulty

☐ 5. I was unable to do anything at all

7. Can you climb stairs to the next floor of a house?

☐ 1. No difficulty

☐ 2. Slight difficulty

☐ 3. I had to rest at least once

☐ 4. I could only climb the stairs with help

☐ 5. I was unable to climb the stairs

8. Do you have problems with dressing?

☐ 1. No difficulty

☐ 2. I can dress myself with slight difficulty

☐ 3. I can dress myself with moderate difficulty

☐ 4. I sometimes need help to dress myself

☐ 5. I cannot dress myself unaided

9. How difficult has it been for you to bend?

☐ 1. No difficulty

☐ 2. Slight difficulty

☐ 3. Moderate difficulty

☐ 4. Great difficulty

☐ 5. I am unable to bend down

10. How much has your walking been limited?

☐ 1. Not limited

☐ 2. Slightly limited

☐ 3. Moderately limited

☐ 4. Very limited

☐ 5. I am unable to walk

11. How difficult has it been for you to visit friends or relatives?

☐ 1. No difficulty

☐ 2. Slight difficulty

☐ 3. Moderate difficulty

☐ 4. Great difficulty

☐ 5. I have been unable to visit friends or relatives

12. Have you felt downhearted?

☐ 1. No

☐ 2. Rarely

☐ 3. Sometimes

☐ 4. Often

☐ 5. Always

13. Are you hopeful about your future?

☐ 1. Always

☐ 2. Often

☐ 3. Sometimes

☐ 4. Rarely

☐ 5. No

14. Have you felt frustrated?

☐ 1. No

☐ 2. Rarely

☐ 3. Sometimes

☐ 4. Often

☐ 5. Always

15. Have you been afraid of falling?

☐ 1. No

☐ 2. Rarely

☐ 3. Sometimes

☐ 4. Often

☐ 5. Always

16. Have you been afraid of sustaining a fracture?

☐ 1. No

☐ 2. Rarely

☐ 3. Sometimes

☐ 4. Often

☐ 5. Always

## Pre-publication history

The pre-publication history for this paper can be accessed here:

http://www.biomedcentral.com/1471-2458/11/775/prepub
